# Epigenetic Regulation by Heritable RNA

**DOI:** 10.1371/journal.pgen.1004296

**Published:** 2014-04-17

**Authors:** Reinhard Liebers, Minoo Rassoulzadegan, Frank Lyko

**Affiliations:** 1 Division of Epigenetics, DKFZ-ZMBH Alliance, German Cancer Research Center, Heidelberg, Germany; 2 University of Nice Sophia Antipolis, Nice, France; 3 Inserm UMR1091, CNRS UMR7277, Nice, France; Queensland Institute of Medical Research, Australia

## Abstract

Genomic concepts are based on the assumption that phenotypes arise from the expression of genetic variants. However, the presence of non-Mendelian inheritance patterns provides a direct challenge to this view and suggests an important role for alternative mechanisms of gene regulation and inheritance. Over the past few years, a highly complex and diverse network of noncoding RNAs has been discovered. Research in animal models has shown that RNAs can be inherited and that RNA methyltransferases can be important for the transmission and expression of modified phenotypes in the next generation. We discuss possible mechanisms of RNA-mediated inheritance and the role of these mechanisms for human health and disease.

Our basic understanding of transgenerational inheritance relies on observations made by our ancestors generations ago. Gregor Mendel's 1860s pioneering studies of pea plant crosses and breeding represent the foundation of what is known about inheritance mechanisms to this day. By crossing pea plants with pure-breeding, i.e. homozygous, characteristics and later also heterozygotes in different combinations, Mendel was able to propose two basic principles, or laws, which apply to sexually reproducing, diploid organisms: The first is the law of segregation, which states that the two alleles of a given trait from each parent segregate into two gametes, which are then passed down to the offspring. This law was confirmed in later studies on meiosis and ensures genomic stability by maintaining ploidy while allowing genetic diversity through new and random combinations of alleles of the same gene. Mendel further observed that different alleles of a trait (such as pea colors) do not blend, but either dominate or recess, allowing only one defined trait to be expressed at a time. Mendel's second law, the law of independent assortment, states that separate traits (for example, pea color and shape) are inherited independently from one another. This principle allows a vast variety of combinations of traits and thus ensures genetic diversity among the offspring.

As a result of decades of research in the framework of Mendelian genetics, we now know that DNA is the main carrier of genetic information from one generation to the next. Methods for genetic analysis have evolved considerably and now allow the deciphering of entire genomes. DNA microarrays and next-generation sequencing have made it possible to identify millions of genetic variants, such as single nucleotide polymorphisms and copy number variants, in thousands of individuals. Currently, large-scale genome-wide association studies (GWAS) are used to unravel highly complex genotype–phenotype relationships and represent a sophisticated conceptual development that is based on Mendelian genetics [1].

Genetic information is organized in higher order structures, which consist of DNA, proteins, and RNA [2]. Together, these factors modulate gene expression and have defined the field of epigenetics [3], [4]. Some of the best-known epigenetic mechanisms include chemical modifications of DNA and histone proteins and regulate the expression of genetic information by rendering the respective regions more or less accessible to the transcriptional machinery [5], [6]. Dynamic epigenetic regulation allows for phenotypic changes of an organism, for example, during development or in response to external stimuli. Epigenetic regulation can be highly dynamic, which is exemplified by the complex reprogramming of DNA methylation patterns during early mammalian embryogenesis and cellular differentiation [7]. DNA methylation patterns are erased in primordial germ cells and preimplantation embryos and then become re-established during the later stages of embryogenesis. These processes are critical for establishing the totipotent state of embryonic stem cells and for determining cellular identity [8], [9]. In addition, DNA methylation has also been suggested to have adaptive functions and may facilitate the plasticity of gene expression patterns. This is exemplified by several studies that have linked environmental or nutritional changes to altered DNA methylation [10], [11]. However, the functional significance of epigenetic mechanisms for adaptive phenotypic changes remains to be established. In this review, we discuss recent discoveries in the field of RNA-mediated inheritance that may shed light on the mechanisms of non-Mendelian transgenerational transmission of phenotypes, and the roles that these mechanisms may play for human health and disease.

## RNA-Mediated Non-Mendelian Inheritance

**Figure 1:**
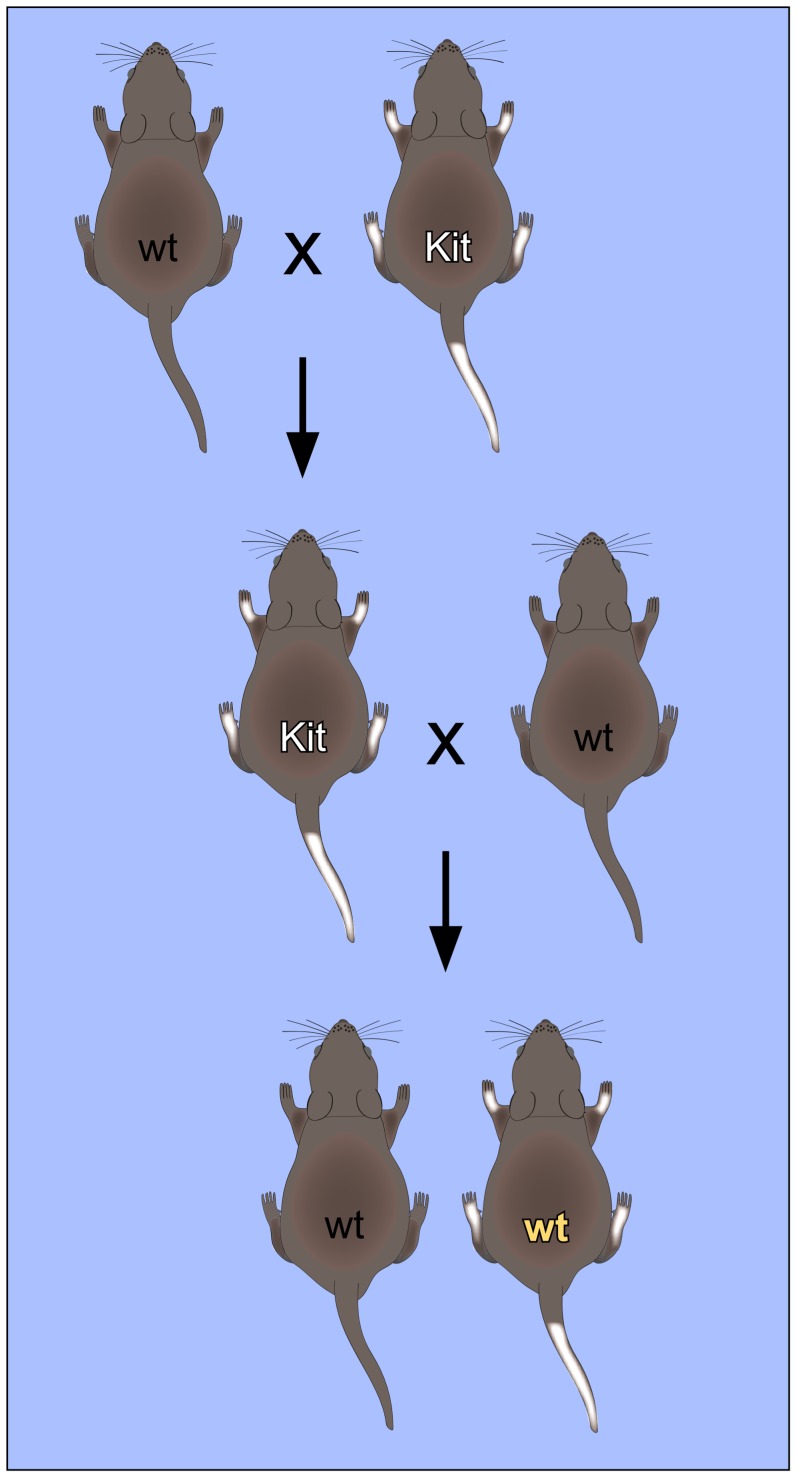
Non-Mendelian inheritance of mouse paramutations. The “white-spotted” Kit^tm1Alf/+^ mouse phenotype provides an important paradigm for RNA-mediated non-Mendelian inheritance. Mating of heterozygous Kit^tm1Alf/+^ (Kit) mice with wild-type (wt) mice results in Kit^tm1Alf/+^ (Kit) offspring with characteristic white tails and feet. When these mice are again mated with wild-type mice, a fraction of the offspring retains the “white-spotted” phenotype, even with a wild-type genotype. This phenotype could also be induced by microinjection of RNA into fertilized oocytes, which suggests that RNA plays an important role in the mechanism of inheritance.

Transgenerational epigenetic inheritance has been described in various systems and detailed reviews on this topic have been published recently [12], [13]. In many cases, the corresponding inheritance patterns can be explained by classical genetic or epigenetic mechanisms [13]. However, it has also been suggested that heritable extragenomic factors, such as RNA, may be involved in this phenomenon [12]. In this review, we will focus on the importance of RNA in non-Mendelian inheritance. This is best illustrated by observations that describe the inheritance of paramutation phenotypes in mice (Figure 1).

The first mouse model for a non-Mendelian mode of heredity was the “*Kit* paramutation” which describes a stable modification of *Kit* gene expression. *Kit* encodes a tyrosine kinase receptor with roles in developmental processes including hematopoiesis, germ cell differentiation, and melanogenesis. A homozygous deletion of *Kit* is lethal, whereas heterozygotes carrying one allele inactivated by a *LacZ* insertion (*Kit^tm1Alf/+^*) show a white-tail phenotype [14]. Interestingly, the white-tail phenotype of heterozygous parents is maintained in their genetically *Kit^+/+^* progeny and in subsequent crosses with wild type partners (Figure 1). These genotypically wild-type but phenotypically mutant mice are termed “paramutants”. First insight into the underlying mechanism came from a powerful assay based on the microinjection of RNA from the parent mouse or synthetic oligoribonucleotides into the pronuclei of fertilized mouse eggs. When injected with sperm RNA of the *Kit* heterozygote or with an RNA fragment derived from the *Kit* transcript, a heritable epigenetic change was induced and a considerable fraction of mice showed the white-tail Kit* phenotype [14]. Similarly, microRNAs that were known to target kit mRNA were also very active in the induction of the paramutant phenotype, presumably by inducing the generation of short noncoding *Kit* RNAs in early embryos [14].

Comparable epigenetic variations were subsequently generated at other loci by microinjection of microRNAs and transcript fragments. Heart hypertrophy could be induced by miR-1 injection and caused an increased expression of the key effector *Cdk9* in cardiomyocyte precursors [15]. Similarly, when miR-124 or fragments of its *Sox9* target transcript were injected, this resulted in *Sox9* overexpression during the first embryonic stages, increased proliferation of embryonic stem cells, increased body sizes during postnatal development, and twin pregnancies [16]. In all three cases, the modified phenotype was associated with an increased rate of transcription of the target locus, thus suggesting the induction of long-term transcriptional activation by fragments of the transcript and/or by cognate microRNAs. Furthermore, these experiments also identified RNA molecules carried by the sperm as the transgenerational vectors of paternal inheritance. Finally, these examples shared three characteristics that clearly distinguished them from genetic mutations that are transmitted according to the Mendelian rules: (i) paramutations could be induced at far greater frequencies; (ii) although eventually reversible, the changes were transmitted both paternally and maternally for three or more generations in crosses with wild-type partners and with close to 100% efficiency; and (iii) paternal inheritance was related to the presence of spermatozoal transcripts of the target gene and/or of the cognate microRNAs.

It is important to notice that these experiments identify RNAs as necessary but certainly not sufficient for the inheritance of phenotypes. The observed sequence specificity for the induced phenotype and the maintenance of the phenotype for three generations make it reasonable to envisage a mechanism that involves a targeted modification of the corresponding genomic locus. There are several examples illustrating locus-specific modulation of gene expression by small RNAs in the mouse [17]–[19], and small RNAs with homology to the target locus are efficient inducers of paramutant phenotypes. However, only a small fraction of genetic loci appears to be paramutable, and the defining features of paramutable loci remain to be identified. Further experimental approaches are needed to define the underlying mechanisms and to directly identify the corresponding regulatory RNAs.

## Heritability of RNAs

**Figure 2:**
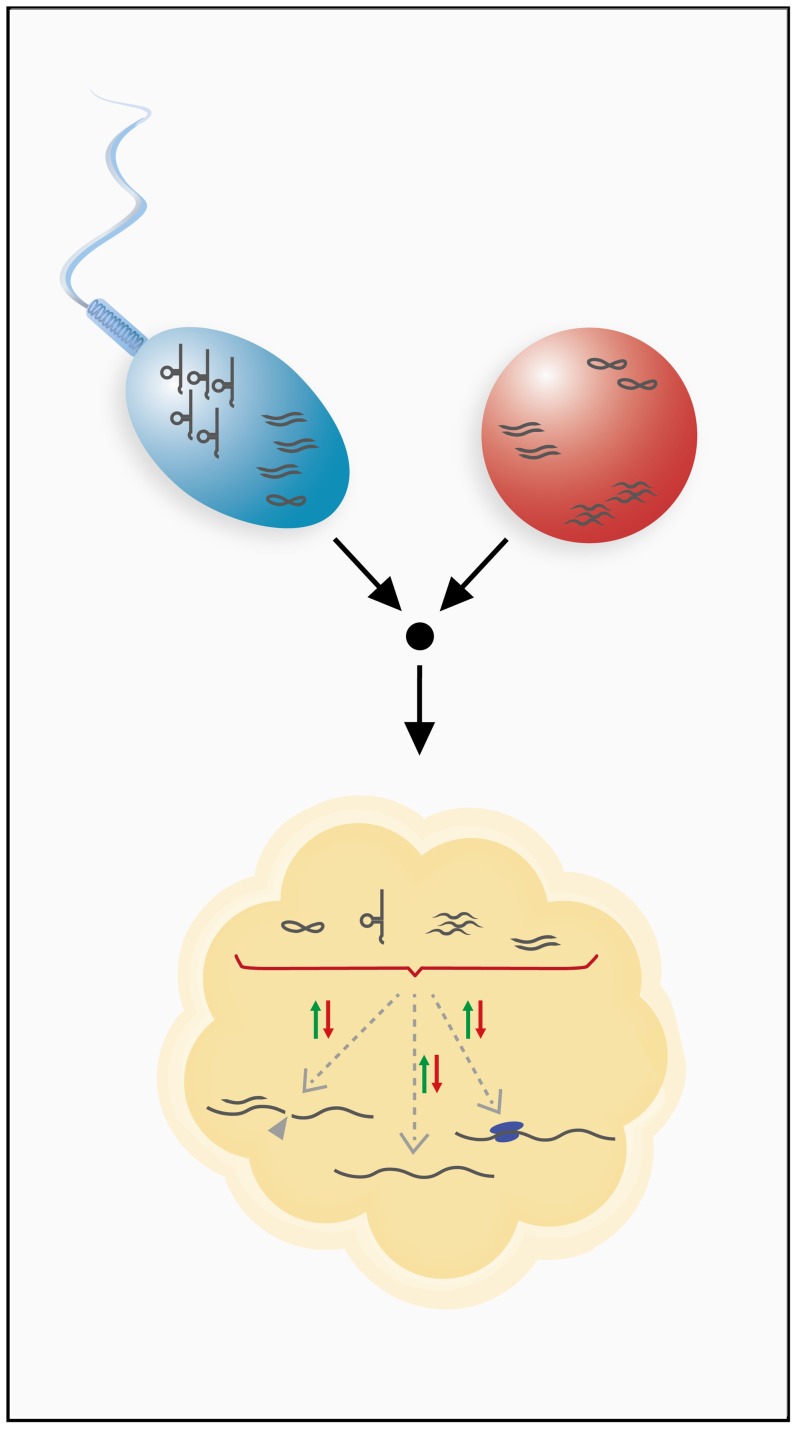
Inheritance of RNA through the male and female germ lines. Sperm and ovum can contribute various classes of RNAs to the developing embryo. Germ line transmission has been shown for mRNAs, small regulatory RNAs, and tRNA fragments. Inherited RNAs are hypothesized to have gene regulatory functions in the developing embryo and could utilize different mechanisms of gene regulation to achieve this. These RNAs might modulate transcript stability, influence transcriptional or translational processes, or possibly engage in other, unknown regulatory pathways.

In order to be heritable, RNAs must be present within gametes of males or females, or in both. Even though both spermatozoa and ova are considered transcriptionally silent [20], [21], several studies have shown that a complex and diverse set of RNAs is present in germ cells of both sexes, as well as in early embryos [22]–[24]. In addition, it has also been shown that spermatozoa are in fact capable of shuttling RNAs into the oocyte as part of fertilization [25]. Together, these findings suggest that a fertilized egg is initially equipped with a diverse and complex RNA “cache” [26] which it inherited from the male and female germ lines (Figure 2).

Because gametes are transcriptionally quiescent, such an RNA cache must be sufficiently stable to last the lifetime of a gamete. Indeed, several oocytic and zygotic transcripts have been shown to contain specific sequence motifs for regulation of stability, which suggests the involvement of regulating factors such as RNA-binding proteins or antisense RNAs [27]. In addition to these sequence motif-related mechanisms, post-transcriptional RNA modifications, such as cytosine-5 methylation, are known to stabilize RNAs [28] and have, in fact, been shown to be present on spermatozoal RNAs [29]. Stabilization of inherited RNAs may also be required during the maternal-to-zygotic transition (MZT), which involves extensive remodeling of RNA profiles. Interestingly, it was shown that specific RNAi effectors that are capable of both destabilizing and stabilizing certain transcripts during the MZT are, in fact, essential for the MZT [30]. Because different classes of small RNAs play a key role in the MZT [31], [32], inherited small noncoding RNAs could be involved in the remodeling of RNA profiles during early embryogenesis (Figure 2).

Several studies have suggested that miRNAs can be inherited, thus providing a possible mechanism for the transgenerational inheritance of altered phenotypes [13]. Indeed, miRNAs represent an intriguing example for a class of small regulatory RNAs with a well-established role in the stabilization of developmental gene expression programs [33]. Another possible mechanism could be provided by the newly discovered circRNAs, which represent stable RNA molecules that can have marked effects on the transcriptome composition through the sequestration of miRNAs and their effector proteins [34], [35]. While the heritability of circRNAs remains to be shown, their longevity and their ability to function as “miRNA sponges” would represent a powerful mechanism for the modulation of transcriptional programs in the developing embryo.

Another class of small regulatory RNAs with a well-established heritability is provided by siRNAs [13]. Endogenous siRNAs are present in mouse germ cells of both sexes [17]–[19], [36], and should thus be heritable, in principle. In Drosophila, endogenous siRNAs can be associated with chromatin through interactions with DCR2 and AGO2 [37], which provides a candidate mechanism for developmental gene regulation by heritable siRNAs. Similarly, long noncoding (lnc) RNAs are also capable of scaffolding protein complexes and recruiting chromatin modifiers to specific sites in the genome, thereby guiding epigenetic changes to specific loci [38]. Finally, it has also been reported that more than half of the small RNAs in mature mouse sperm are tRNA fragments [39]. tRNA fragments have the capacity of altering gene expression by functioning as siRNA mimics [40], [41] or inhibitors of translation initiation [42], thus providing additional potential mechanisms for heritable modulation of developmental gene expression. It should be noted that a substantial amount of further research will be required to understand the precise mechanisms of gene regulation by inherited RNA molecules.

## RNA Methylation: An Epigenetic Mark for RNA-Mediated Inheritance?

RNA can be modified in a diverse and complex manner, but the function of these modifications is only beginning to be explored. For example, adenine-6 methylation (m6A) represents the most prominent modification of mammalian mRNAs. The availability of m6A-specific antibodies provided an excellent opportunity to identify methylated RNAs by immunoprecipitation and sequencing of mRNA fragments. Indeed, two recent studies have identified adenine methylation marks in several thousand mRNAs, with a distinct enrichment in the vicinity of stop codons and in internal exons [43], [44]. Further analyses indicated an association between m6A and RNA splicing [43] and/or microRNA binding [44]. While important mechanistic details remain to be investigated, these data clearly suggested that RNA methylation may have a role in the regulation of gene expression.

Cytosine-5 methylation (m5C) represents another prominent modification of RNA, which can be detected at single-base resolution by bisulfite sequencing [45]. Early transcriptome-wide mapping studies suggested that m5C is prevalent in tRNAs but can also be found in other RNA species [46]. Indeed, the two known cytosine-5 RNA methyltransferases, NSUN2 and DNMT2, were found to be primarily associated with tRNA [47]. Interestingly, NSUN2 was also found to methylate mRNA, rRNA, and several lncRNAs [47]. A particularly interesting example is provided by vault ncRNAs, which can be processed into small RNAs that regulate gene expression [48]. NSUN2-dependent methylation of specific vault target sites has been detected by independent approaches [47], . Notably, loss of vault RNA methylation in NSun2-deficient mice caused aberrant vault processing into Argonaute-associated small RNAs, as well as aberrant expression of several mRNAs that are putative targets of vault-derived small RNAs [49]. These findings provided the first mechanistic insight into the role of RNA methylation in gene regulation.

Additionally, the regulatory role of RNA methylation in gene expression has also been investigated through the functional characterization of the DNMT2 RNA methyltransferase. DNMT2 is closely related to the established DNA methyltransferases and key epigenetic regulatory enzymes DNMT1 and DNMT3. However, DNMT2 does not methylate DNA [50], but rather shows a pronounced substrate specificity towards a highly defined set of tRNAs [47], [51], [52]. DNMT2-mediated methylation has been shown to protect substrate tRNAs against endonucleolytic cleavage [52]. This effect has been further investigated in mice that lack both Dnmt2 and NSun2, where tRNA hypomethylation was associated with decreased tRNA levels and a significant reduction in protein translation rates [53]. More recently, it has also been shown that Dnmt2 is required for efficient Dicer-2 dependent siRNA pathway activity in Drosophila [54]. Dnmt2 plays an important role in the generation of tRNA fragments [52]. These fragments are abundant in eukaryotic cells and are known to affect the efficiency of small RNA silencing [40], suggesting that they affect gene expression by competing with endogenous small RNAs for the effector proteins of the siRNA pathway [41], [54].

Finally, it has been shown that the inheritance of paramutant mouse phenotypes requires an intact *Dnmt2* gene [29], thus suggesting a role for Dnmt2-mediated RNA methylation in RNA-dependent inheritance. In addition, while microinjection of the *Kit* RNA fragments into wild-type fertilized eggs induced up-regulation and possibly methylation of the Kit transcript, these processes could not be observed in Dnmt2-deficient mice. Importantly, corresponding Kit genomic DNA sequences remained unmethylated in wild-type as well as in Dnmt2-deficient mice [29], which further suggested a role of RNA methylation in the modulation of gene expression during early stages of development. Compared to other tissues, Dnmt2 is highly expressed in mouse and human testes and ovaries [55], [56], and Dnmt2-dependent tRNA methylation has been demonstrated in mouse sperm [29]. This raises the possibility of transgenerational inheritance of RNA methylation signals through the male germ line. The precise mechanism for RNA methylation-dependent inheritance of acquired phenotypes will be an important topic for future research. The analysis of modified RNAs from a limited amount of tissue, such as from fertilized eggs, will require low input methods or even single cell sequencing. This may be achieved by novel sequencing technologies, such as single-molecule real-time (SMRT) sequencing [57], or nanopore sequencing [58]. Another important area of research will be the identification of proteins that interact with heritable RNA. This could be facilitated by improved methods that allow the sequencing of crosslinked and immunoprecipitated RNAs, such as iCLIP [59] and HITS-CLIP [60].

## The Significance of RNA-Mediated Inheritance for Human Disease

Interestingly, several recent findings suggest that the mechanisms of RNA-mediated inheritance might be relevant for human health and disease (Table 1). For example, variants in RNA methylating as well as demethylating factors have been genetically associated with pathological phenotypes, such as obesity and intellectual impairment. Variants of the FTO gene, a nonheme FeII/α-KG-dependent dioxygenase that catalyzes the demethylation of m6A in RNA [61], have been associated with high body mass index, risk of obesity, and type 2 diabetes [62]. While the disease-associated genetic variants are characterized by Mendelian inheritance, these findings suggest that altered RNA methylation patterns can have a considerable pathophysiological relevance. Indeed, mutations in another RNA modification enzyme, the NSUN2 methyltransferase, have been shown to cause autosomal recessive intellectual disability [63], [64]. A diagnostic tRNA substrate of NSUN2 appeared clearly hypomethylated in dermal fibroblasts from mutation carriers [65], thus suggesting that RNA hypomethylation is involved in the molecular disease pathology. Interestingly, mouse NSun2 is highly expressed in testis and required for testis differentiation [66]. In addition, NSun2-dependent tRNA methylation is present in mouse sperm [29] and is thus potentially heritable through the male germ line.

**Table 1:** Potential links between heritable RNAs and human health and disease.

Disease/Effect	Cause	Inheritance	References
Obesity, type 2 diabetes	FTO gene variants	Genetic variants in a m6A RNA demethylase gene	[54], [55]
Intellectual disability	NSUN2 mutations	Genetic mutations in a tRNA methyltransferase gene	[56]–[58]
Reduced lifespan	Food surplus during early adolescence	Non-Mendelian, not understood	[61], [62]
Neonatal adiposity and poor overall health	Restricted food supply during pregnancy	Non-Mendelian, not understood	[63]–[65]

Furthermore, RNA-mediated inheritance could also provide an explanation for the missing heritability problem of complex human diseases. The “missing heritability” phenomenon was defined after even extensive GWAS failed to identify major risk factors for complex diseases [67]. This may in part be explained by methodological limitations of current GWAS approaches. The range and sensitivity of the assays that are applied for detection of phenotypes and genomic variants are not always sufficient for a conclusive analysis. In addition, noncoding RNAs have not been sufficiently incorporated into GWAS. Alternatively, however, missing heritability may also be explained by additional, non-Mendelian inheritance mechanisms. A prominent example is provided by epidemiological studies of the Överkalix parish in northern Sweden, which was exposed to fluctuating phases of food supply. A detailed analysis of this cohort indicated that a surplus of food supplies during early adolescence of paternal grandfathers resulted in decreased life span of grandchildren [68], [69]. Even after more than a decade of research, no genetic or epigenetic variations have been identified that could explain the inheritance of this phenotype. Similarly, results from the Dutch Hunger Winter Families cohort [70] showed that a hunger period during pregnancy can lead to poor health of female offspring in the F1 and F2 generations [71], [72]. This inheritance pattern has been associated with DNA methylation changes in the human IGF2 gene, and several other studies have provided evidence suggesting that altered DNA methylation patterns may link nutritional exposures in the parental or grandparental generation to human health and life span [73]. However, the effect sizes of the reported environment-induced DNA methylation differences appear to be very small in humans and in rodent models [74]–[76], which again raises the possibility that additional mechanisms could be involved. RNA-mediated inheritance could provide an attractive mechanism that allows a rapid adaptation to changing environmental conditions without affecting the genetic makeup of an organism.
